# Static and dynamic failure mechanisms of circular granite under the condition of water-heat cycles

**DOI:** 10.1038/s41598-021-85314-2

**Published:** 2021-03-15

**Authors:** Chun Wang, Xin-ru Li, Mei-zhi Xie, Zu-qiang Xiong, Cheng Wang, Huai-bin Wang, Shuai-fei Zhan, Ya-chao Hu

**Affiliations:** 1 School of Energy Science and Engineering, Henan Polytechnic University, Jiaozuo, 454000 Henan China; 2 Guangxi Key Laboratory of Disaster Prevention and Engineering Safety, Guangxi University, Nanning, 530029 Guangxi China; 3 College of Civil Engineering and Architecture, Guangxi University, Nanning, 530029 Guangxi China; 4 State and Local Joint Engineering Laboratory for Gas Drainage and Ground Control of Deep Mines, Henan Polytechnic University, Jiaozuo, 454000 Henan China; 5 The Collaborative Innovation Center of Coal Safety Production of Henan, Jiaozuo, 454000 Henan China

**Keywords:** Energy science and technology, Engineering, Materials science

## Abstract

Based on the engineering environment where rocks surrounding wellbores in energy storage areas are influenced by high temperature, cool and hot water, thermal stress etc. in the exploitation of hydrothermally geothermal energy, the experimental study on mechanical properties of ring granite under the static and dynamic loads in the water-heat condition was performed. The experimental results showed that when the ring granite was influenced by the inner diameters, heating temperatures, curing temperatures and heat recovery cycle times, the impact load-strain curves were nonlinear. However, the concave stages, platform stages and cliff-like drop stages appeared in the load-strain curves under the static loads. The radical peak loads decreased exponentially with the growth of the damage factors and the dynamic peak loads were far greater than the static peak loads. By analyzing the damage cracks and broken fragments, it was found that under the static and dynamic radical loads, the cracks generated in the ring specimens were tensile cracks and the failure mode was tensile failure. However, the dynamic failure was more aggressive than the static failure. Then, the apparent deformation modulus was defined to describe the deformation characteristics of ring granite before the radical peak loads. And it is found that the variation law of dynamic apparent deformation modulus is more dispersed than the changes of static apparent deformation modulus. Finally, based on the deformation and failure characteristics of ring granite obtained from the tests, the static and dynamic failure criteria considering whether the cracks along the loading direction were generated in the inner ring wall were deduced and verified by the corresponding tests.

## Introduction

As the society and economics developed, energy environmental problem has been valued high by many countries around the world, it is extremely urgent to effectively solve the problem of energy and environment coordinated development^1,2^. Geothermal energy is a clean and renewable energy with good stability, better safety, wide range of applications, and so on^3,4^. It will be an effective way to alleviate the coordinated development of energy and environment if the sustainable and efficient exploitation of geothermal energy can be realized. Currently, collapse accidents always occur in mining wells and recharge wells during the exploitation of hydrothermal geothermal energy, which delays the sustainable exploitation of geothermal energy. The appearance of the accident phenomenon is attributed to the lack of investigations on mechanical characteristics, failure modes and criteria of rocks surrounding wellbores.

Scholars in the field of rock mechanics and geothermal new energy have also carried out preliminary studies on mechanical characteristics of rocks surrounding a high-depth wellbore^5–7^, e.g., damage failure mechanism and criteria of deep rocks under conditions of coupled static and dynamic load, high temperature, dry–wet cycle. By analyzing the mechanical characteristics of deep rocks under coupled static and dynamic load, it was found that the dynamic tensile strength of rocks first increased and then decreased with increasing the axial static load, and the failure mode is mainly frictional damage of tension and shear mixture^8–10^. Based on the correlation among static compressive strength, tensile strength and shear strength, Huang S.^11–13^ et al. considered that Mohr–Coulomb criterion coordinating the loading rate can be used to predict the dynamic impact tensile failure of deep rocks by analyzing the relationship among dynamic compressive strength, tensile strength and shear strength. By studying the static and dynamic damage characteristics of deep rocks in high temperature environment, it was seen that the thermal damage delayed the rock crack process and weaken the brittleness^14–16^, and it was also found that static and dynamic compressive strength, tensile strength and shear strength decreased with the increasing temperature^17–20^. Part of scholars established the constitutive model of thermal damage in deep rocks based on damage mechanics and the influence of high temperature on the rock, which improve the accuracy of predicting the rock failure in the high-temperature condition^21–23^. The scholars in field of rock mechanics carried out exploratory studies on the damage failure mechanism of deep rocks affected by cool and hot water. It was found that the compressive strength of dry rocks may be larger than that of saturated rocks under higher loading rates, dynamic elastic modulus of rocks was larger than static elastic modulus of rocks under saturation conditions^24,25^. However, the relationship was opposite under dry conditions by comparing the static and dynamic compressive strength of dry and saturated rocks^26–28^. Moreover, based on damage mechanical characteristics of deep rocks in the environment that cool and hot water cycled, Wang^29,30^ et al. established the constitutive model of force-heat-water coupling of deep rocks to reveal the influence of temperatures and water on the deformation of rock. By comparing the studies mentioned above, it was seen that high temperature, dynamic disturbance and water were taken into account in the investigation on dynamic and static failure characteristics of deep rocks. However, the studies on dynamic and static characteristics of deep rocks in the engineering environment that the exploitation of hydrothermal geothermal energy experienced are not enough, especially the influence of the cross section of geothermal wellbore, medium water and cycle heat recovery times are ignored.

In order to make up for the lack of researches on the damage characteristics of rocks surrounding geothermal wellbores, circular granite specimens were used to carry out the experimental study on static and dynamic mechanical properties under the assumptions that the maximum load that the rock surrounding wellbores was the horizontal stress and travelled along the radical direction of the wellbore cross section. The experiment aimed at revealing deformation characteristics, damage process, final failure modes and features of circular granite, and establishing corresponding failure criteria to provide theoretical reference for preventing the damage failure of rocks surrounding wellbores in geothermal energy storage areas.

## Preparation for water-heat cycle test

### Specimen preparation

The granite used in the test was taken from the Liuzhuang granite mine, which was located in Biyang County, Henan Province, China. To reveal the static and dynamic failure characteristics of circular granite under water-heat cycle, two types of specimens were drilled from granite blocks with good integrity and homogeneity. One type was cylindrical specimens with a height of 30 mm and a diameter of 50 mm. Another type was concentric ring specimens with the same size except for inner diameters, which were divided into four levels: 6 mm, 12 mm, 18 mm and 22 mm. At the same time, to accurately detect the mechanical characteristics parameters of granite specimens under radial static and dynamic load, both ends of specimens were grinded and polished according to Rock mechanics test sample treatment requirements to ensure that their non-parallelism and non-verticality were no more than 0.02 mm.

### Experimental apparatus

The test system consisted of a GCTS multifunctional rock mechanics test system, a SHPB impact mechanics test system and VIC-3D non-contact full-field strain measurement system. GCTS was used to carry out static load compression tests on circular granite. And its maximum load range was ± 1500KN, the measure precision was ± 0.25%, displacement loading rate range was 0.01 mm/min ~ 700 mm/min. SHPB was used to carry out the radical impact compression test of circular granite. And the lengths of incident and transmitted bar were 3 m, and the bullet was 0.4 m long. VIC-3D, which consisted of a super-speed camera, lighting system, trigger, synchronization control system and computational analysis system, was used to seize the failure process of ring granite during the static and dynamic test.

### Experimental scheme

Rocks surrounding wellbores in the energy storage areas were in the conditions of high temperature, medium water at different temperature and thermal stress impact during the exploitation of deep geothermal energy. The damage failure mechanism was closely linked to the factors mentioned above. In order to reveal the damage failure mechanism of rocks surrounding wellbores in the energy storage areas, the experimental study combined with the engineering environment that the rocks surrounding wellbores experienced in the deep geothermal energy mining was carried out under the assumption that the horizontal stress was the main factor leading to the failure of rocks surrounding wellbores in energy storage areas. In the static and dynamic mechanical test, high-temperature treatment, water curing at set temperatures, heat-curing times were exported to simulate the influence of high temperature, medium water and cycle heat recovery times on the rocks surrounding wellbores in energy storage areas, the impact load was used to simulate the thermal stress effect generated by dynamic disturbances. The displacement loading rate was 0.02 mm/min in the static test. In order to ensure that the ring specimens were broken after one impact applying the impact load equal to 0.2 MPa, the pressure value leading to impact failure on Brazilian discs at room temperature, in the dynamic impact test. Based on the engineering environment that rocks surrounding wellbores in energy storage areas, five levels were chosen from the variable factors involved in the test, the specific scheme is shown in Table 1. Meanwhile, in Table 1, high temperature treatment means the specimens were heated at a heating rate of 2 °C/min until the temperature arrived at set temperature, then the temperature were kept constant for 2 h; water curing means the high-temperature specimens were cooled down to a set temperature under natural condition, and then the specimens were immersed in water at the same temperature for 1 h; one cycle heat recovery means including one high temperature treatment and one water temperature curing.

**Table 1:** Static and dynamic test variable factors of circular granite under water-heat cycles conditions.

Numerical order	Inside diameter/mm	High temperature Treatment/°C	Curing Temperature/°C	Cycles/times
1	0	100	10	1
2	6	250	25	3
3	12	400	40	5
4	18	550	55	7
5	22	700	70	9

1. High temperature treatment: The specimens were heated at a heating rate of 2 °C/min until the temperature arrived at set temperature, then the temperature were kept constant for 2 h. 2. Water curing: The high-temperature specimens were cooled down to a set temperature under natural condition, and then the specimens were immersed in water at the same temperature for 1 h. 3. One cycle heat recovery: It includes one high temperature treatment and one water temperature curing.

## Experimental results

### Deformation characteristics of circular granite

**Figure 1:**
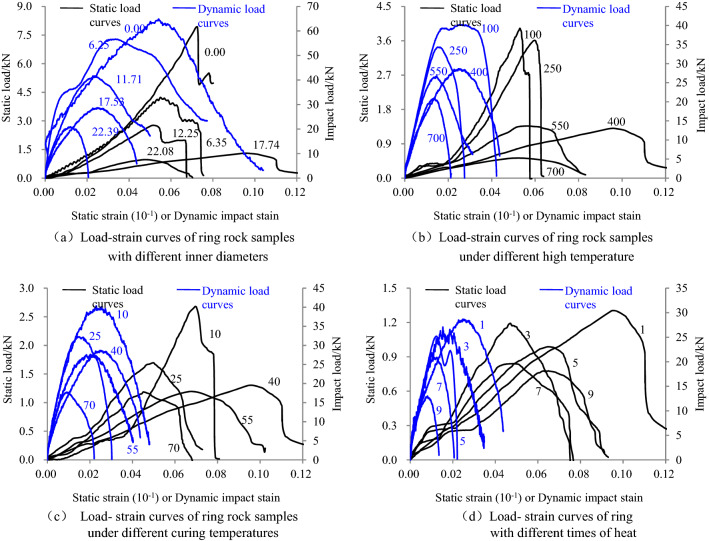
Load-strain curves of circular granite under the condition of water-heat cycles (The figures in (**a**) represent the inner diameter of the circular granite samples, and their units are mm. The figures in (**b**) represent the high temperature at which the circular granite samples were processed, and their units are °C. The figures in (**c**) represent the water temperature for curing the circular granite samples, and their units are °C. The figures in (**d**) represent the cycle heat recovery times of circular granite samples, and their units are time. The inner diameter of the rock samples are all about 18 mm in (**b**–**d**)).

The failure mechanism of ring specimens can be indirectly revealed by comparing and analyzing deformation characteristics of ring granite under static and dynamic load. The static and dynamic load-strain curves of ring specimens with different inner diameters, high temperature, curing temperature and heat recovery times are shown in Fig. 1.

Figure 1 shows that two types of static load-strain curves appear in pre-peak and post-peak stage. The curves in pre-peak stages are upper concave stages-straight stages and straight stages-platform stages-approximate straight stages, and the latter appears frequently in the curves. The curves in post-peak stages are cliff-like drop stages-platform stages-cliff-like drop stages and nonlinear drop stages. In the pre-peak stages, the upper concave stages appeared because inner diameters of ring specimens decreased rapidly along the loading direction under radical loads, and then, the inner diameters reached the critical value, the elastic deformations were generated in ring specimens under compressive loads. At that period, the straight stages appeared in the load-strain curves. High temperatures and cycle heat recovery damaged the inner structure of ring specimens, which led to the reduction of the compressive capacity, the ring specimens were easier broken under radical loads. It was shown as first short straight stages and then platform stages in the load-strain curve. The platform stages appeared because ring specimens were broken into two half rings, the contact area between the bearing plate and specimens gradually increased, radical deformations instantaneously increased. When the contact area reached to a certain value, two half rings together resisted the loads, which indirectly strengthened the compressive capacity. At this moment, approximate straight stages appeared again in the curve. Cliff-like drop stages appeared in the post-peak curve because macro-failure was generated in the specimens, the capacity to resist radical loads suddenly dropped. When the ring specimens were broken into two fragments, the radical compression loads were supported by the two half rings, which deferred the instability failure of whole ring structure of specimens, which was shown as the platform stages in the load-strain curves. The loads made deformations of two half rings reach the limit, the brittle failures were generated again, the curves continues to develop in the trend of cliff-like stages. Some damages were accumulated in the interior of ring specimens after high-temperature treatment, water curing and cycle heat recovery, which weakened the brittleness of the whole rings. The failure modes of ring specimens under radical loads were transformed from brittleness failures to plastic failures, which were shown as the nonlinear drop in the post-peak load-strain curves.

The load-strain curves of ring specimens under impact loads were nonlinear in general. Since the impact loads were applied on the ring specimens instantly, the elastic stages were generated before the micro-cracks along the impact direction closed. At the same time, the deformations of inner rings were lagged behind in initial impact stages, which were shown as the short straight stages in the impact load-strain curves. The impact loads promoted the generation, propagation and penetration of micro-cracks inside ring specimens, damage deformations were generated in the inner circle with the increasing impact loads. In turn, when the impact load increases, the strain increment along the impact direction also increases rapidly, which was reflected in the curve is a nonlinear upward trend development. When the impact loads reached the maximum loads that ring specimens can bear, micro-failures were generated in the specimens. Since the impact loading time was short, the hysteretic effect and toughness and delay effect of the ring structure weakened the brittleness of ring specimens and strengthen the plasticity of specimens, which were shown as nonlinear in the post-peak curves.

By comparing static and dynamic load-strain curve, it was found that the influence of inner diameters in rings, heating temperatures, water curing temperatures and heat recovery times on the curves under static compression loads was larger, especially the types of the curves were transformed from the cliff-like drop to the nonlinear drop in post-peak stages. However, the influence on impact load-strain curves was minor, which was shown as nonlinear under different factors in the curves. After further analysis, it was found that the strains of ring specimens along the impact direction under impact loads were 5–10 times as big as the strains under static loads and the result was not affected by inner diameters, heating temperatures, water curing temperatures and heat recovery times. It indicates that the brittleness of ring specimens under impact loads was stronger than that of specimens under static loads and the shorter the radical loading time was, the larger the deformations that the ring specimens could bear were. In other words, the capacity of instant deformations became stronger.

### Peak loads of circular granite

**Figure 2:**
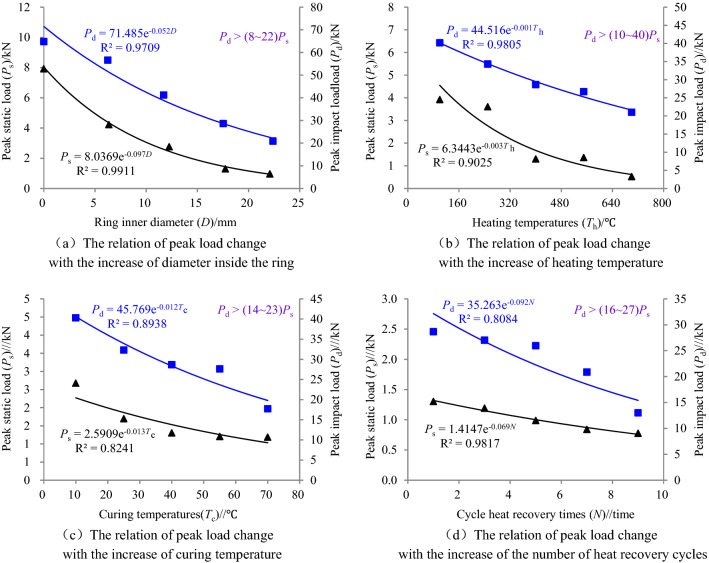
Relationship between peak load and ring inner diameter, heating temperature, curing water temperature and times of heat production cycle (The inner diameter of the rock samples are all about 18 mm in (**b**–**d**)).

When the tensile strength of ring granite under radial loads is calculated by the Brazilian strength formula, the error of calculus results is larger. Therefore, the strength calculated by the formula can not reflect the real capacity to resist the loads of ring structures. Radical compression loads and impact loads were directly detected in the test, which were more precise to reflect the capacity of the ring structure under different environmental conditions. The curves of relationships between static and dynamic peak loads and inner diameters of rings, heating temperatures, curing temperatures and heat recovery times were drawn in Fig. 2.

Figure 2 shows that the static and dynamic peak loads decreased exponentially with the increasing inner diameters of rings, heat recovery times, heating temperatures and curing temperatures. When the inner diameter of ring specimen is larger, the stress effect in the ring wall under radical loads became more noticeable. At this moment, the thickness of rings was smaller, the stress effect that per unit volume could bear became larger, and the rings were more easily broken, which is shown as the decrease of static and dynamic peak loads in Fig. 2a. When the ring specimens were treated under high temperature conditions, the moisture were faster evaporated with increasing temperatures, which led to the pore volume without filling in the rock sample is increased. At the same time, the volume of specimens dilated under the heat, which weakened the structural joint stress of particles in ring specimens. Moreover, the thermal stress of the structure among particles of specimens became stronger as temperatures increased. The old and newly generated cracks appeared and expanded, which led to thermal damages to the ring specimens. It is shown in Fig. 2b that static and dynamic peak loads decreased with increasing heating temperatures due to the reasons mentioned above. Some damage appeared inside the ring specimens after high temperature treatments. When the specimens were placed in water curing at different temperatures, the mobility of water molecules became stronger and they penetrated faster into the damage cracks as water temperatures increased. When the curing time kept constant, the extent of the water saturation increased with the increasing curing temperatures and the extent of the softening became more noticeable. During this period, the capacity of the ring specimens to resist the compressive loads weakened. It is shown in Fig. 2c that the static and dynamic peak loads decreased exponentially with the increasing curing temperatures. During the heat recovery, the damage process of ring specimens was in a continuous cycle, e.g. the ring specimens experienced the cycle process of dehydration, water saturation, heat-expansion and cold-contraction under high temperature and warm-water curing conditions. After each cycle, the extent of damages inside the specimens inevitably increased. It is shown in Fig. 2d that the static and dynamic peak loads decreased with the increasing cycle heat recovery times.

By further comparing analyzing the change rules of static and dynamic impact peak loads under the influence of inner diameters of rings, heating temperatures, curing temperatures and heat recovery times, it was found that the static and dynamic peak loads decreased exponentially, but the value of dynamic loads were far greater than that of static loads, e.g., the dynamic peak loads were 8–22 times, 10–40 times, 14–23 times and 16–27 times as big as the static loads under the conditions of inner diameters of rings, heating temperatures, curing temperatures and heat recovery times respectively. The analysis results appeared because the loading time was longer under static loads, the cracks inside the ring specimens fully penetrated. The final macro-failure was generated in the loading process and the accumulative damage effect was noticeable, which led to the weakening of the capacity of the circular granite samples to resist the loads. However, loading and unloading were instantly completed under the dynamic loads. The internal damage defect of the circular granite sample even loses its power source before it germinates sufficiently, that is, the unloading of external impact load has been completed in the process of internal micro-cracks propagation. Therefore, the dynamic peak loads that ring specimens could bear were far greater than the peak loads under static compression.

### Apparent deformation modulus of circular granite

**Figure 3:**
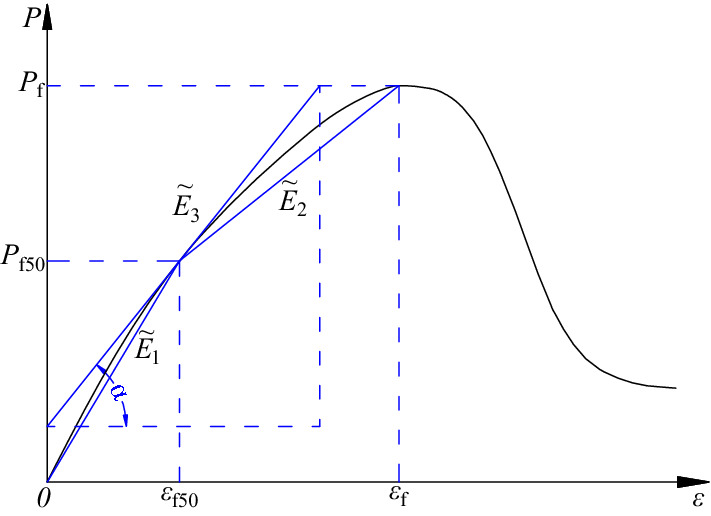
Schematic diagram of the method for defining the apparent deformation modulus of circular granite.

In order to investigate the deformation mechanism of ring granite under the static and dynamic loads before the peak loads, the apparent deformation modulus was defined based on the calculation principle of the elastic modulus of rocks, i.e., the apparent deformation modulus was determined from the load-strain curves of ring granite under static and dynamic loads. For reflecting the deformation mechanism of ring granite in the whole loading stages, 50% of the peak loads was chosen as the demarcation point to reduce the error and discreteness of apparent deformation modulus. Based on the calculation principles of the first kind of secant modulus, the second kind of secant modulus and tangential modulus, the corresponding apparent deformation modulus were defined, and the average value of the modulus mentioned above was taken as the apparent deformation modulus of ring granite. The calculating methods of each parameter are shown in Fig. 3.

According to Fig. 3, the formula to calculate the apparent deformation modulus of ring granite is obtained as follows:

1 $$ \tilde{E} = \frac{1}{3}\left( {\tilde{E}_{1} + \tilde{E}_{2} + \tilde{E}_{3} } \right) = \frac{1}{3}\left( {\frac{{P_{{{\text{f}}50}} }}{{\varepsilon_{{{\text{f50}}}} }} + \frac{{P_{{\text{f}}} - P_{{{\text{f}}50}} }}{{\varepsilon_{{\text{f}}} - \varepsilon_{{{\text{f50}}}} }} + \tan \alpha } \right) $$

where $\tilde{E}$ is the apparent deformation modulus of ring granite, $\tilde{E}_{1}$, $\tilde{E}_{2}$, $\tilde{E}_{3}$ are the first kind of apparent secant modulus, the second kind of apparent secant modulus and apparent tangential modulus respectively.

Based on the methods to define the apparent deformation modulus of ring granite, the curves of static and dynamic apparent deformation modulus with increasing diameters of inner rings, heating temperatures, curing temperatures and cycle heat recovery times were drawn, as shown in Fig. 5.

**Figure 4:**
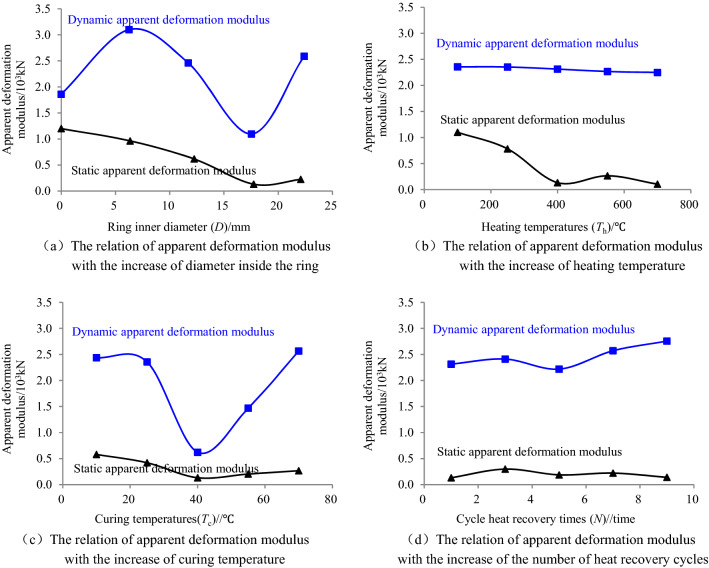
Relationship between apparent deformation modulus and ring inner diameter, heating temperature, curing water temperature and times of heat production cycle (The inner diameter of the rock samples are all about 18 mm in (**b**–**d**)).

As shown in Fig. 4, under the action of radial load, the dynamic apparent deformation modulus of circular granite is greater than the corresponding static apparent deformation modulus. At the same time, the mutability of the dynamic apparent deformation modulus is stronger than that of the static apparent deformation modulus, especially under the two environmental conditions of increasing the inner diameter of circular granite and increasing the curing water temperature. Under the impact load, the whole process of loading and unloading of rock samples is completed instantaneously, which leads to the process of germination, expansion and transiting of tensile crack in circular granite is also completed in an instant. At this time, the rock sample produced macroscopic failure. This phenomenon is reflected in the fact that the impact load borne by the circular granite rock samples is greater than the static compression load, and the impact strain is smaller than the static compression strain, finally resulting in the dynamic apparent deformation modulus greater than the static apparent deformation modulus under the same environmental conditions. In the static test, the increasing rates of static loading were smaller, the inner structure of ring granite was relatively stable, and the generation and expanding of damage cracks were also flat. Therefore, the change rules in ring granite under static loads were more noticeable. At the same time, since the same loading rate by displacement loading was employed in the test, the change rules in the increase of loads were also more noticeable. The regular increase of loads and deformations finally made the deformation of ring granite in the test more able to reflect the real situation of damage evolution inside the ring specimens and it was shown that the apparent deformation modulus decreased with the weakening of the capacity of the circular granite samples to resist static loads. Under the impact loads, the loading and unloading were completed instantly, the deformation of ring granite was lagged behind, and abrupt changes occurred in the propagation of damage cracks inside the ring specimens. Moreover, different size of rock particles made the stress concentration effect more noticeable. These factors eventually lead to the deformation homogenization trend of ring granite became weak. It was shown that the apparent deformation modulus more dispersedly changed under different factors. E.g., various change trends appeared in the deformation modulus of ring specimens with the weakening of the capacity to resist the impact loads in Fig. 4.

## Failure characteristics of circular granite

### Damage process

**Figure 5:**
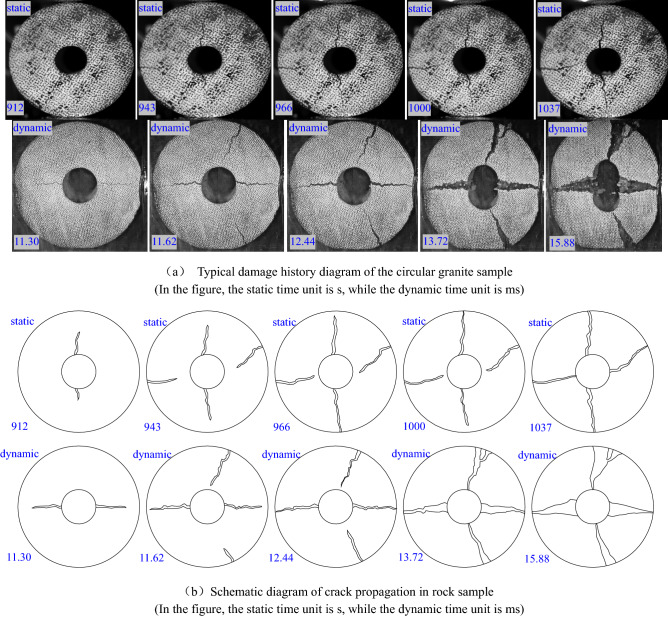
Damage and failure process of circular granite under radial load (The inner diameter of the rock samples are all about 12 mm in (**a**–**b**)).

The damage and failure process of ring granite under static and dynamic loads can effectively reveal the failure mechanism and indirectly reflect the damage failure mechanism of rocks surrounding high-depth wellbores. In order to compare and analysis the damage evolution inside the ring granite under the static and dynamic radical compression loads, a group of typical pictures of crack propagations were shown in Fig. 5a and the diagrams of crack propagations were drawn in Fig. 5b.

Figure 5 shows that the damage cracks initiated from the inner ring wall of ring granite along the loading direction. When the cracks expanding distance reached the half of the ring thickness, the second group of cracks initiated in the vertical loading direction from the outer ring wall. The propagation direction of the two groups of cracks expanded along the radical direction of ring granite until the whole specimen was penetrated. By analyzing the propagation process of two groups of cracks, it is seen clearly that the crack spacing was gradually enlarged, the fracture surfaces were not dislocated, and the bifurcation phenomenon did not occur in the process. Therefore, it was obtained that the cracks generated in ring granite were the tensile cracks caused by the tensile stress effect. It was found that the stress effects generated in each direction of inner ring wall were various in the initial stages of static compression loads and impact loads via the analysis of the pressure applied in ring granite, i.e., the tensile stress effect was generated along the loading direction and the compressive stress effect was generated in the vertical loading direction. Since the compressive strength was far larger than the tensile strength for rocks, the damage cracks first initiated along the loading direction and expanded to the outer side of ring specimens. At the same time, the tensile stress effect appeared in the outer side of ring specimens, but the strength was less than the stress inside the ring granite along the loading direction, the crack initiation in the vertical loading direction lagged behind that along loading direction.

By analyzing the modes of damage cracks under static and dynamic compression loads, it is easy to find that the cracks generated under the impact loads expanded rapidly and the crack spacing was larger. However, the cracks generated under the static loads expanded slowly and the crack spacing was smaller. This phenomenon indicates that the dynamic damage of the ring granite is more severe than the static damage. Further analyzing the spacing between the two sets of cracks, it was found that the fracture surface spacing of static damages in the initiation position and propagation were the same. As for the fracture surface spacing of dynamic damages, the spacing in the initiation position was far larger than the spacing in the propagation position, which revealed that the damage cracks evolution inside the ring specimens under dynamic impact loads lagged behind the impact loads, i.e., it was no enough time to respond for the internal structural damage of ring granite under instant impact loads, so that the damage characteristics were lagged behind in the dynamic loads.

### Failure modes

**Figure 6:**
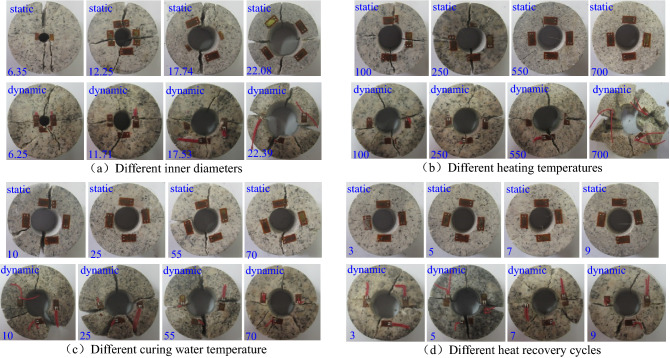
The final failure state of circular granite under radial compression (The figures in (**a**) represent the inner diameter of the circular granite samples, and their units are mm. The figures in (**b**) represent the high temperature at which the circular granite samples were processed, and their units are °C. The figures in (**c**) represent the water temperature for curing the circular granite samples, and their units are °C. The figures in (**d**) represent the cycle heat recovery times of circular granite samples, and their units are time. The inner diameter of the rock samples are all about 18 mm in (**b**–**d**)).

The failure modes of ring granite under static and dynamic loads are also an effective way to reflect its stress characteristics, which can reveal the forms of damage crack propagation inside rock samples. Figure 6 shows the typical failure modes of ring granite in static compression loads or dynamic impact loads under various factors.

Figure 6 shows that the two groups of principal crack zones were generated in the ring granite under the static loads or dynamic impact loads. One group penetrated the ring granite and the fracture surfaces across the center of a ring were generated along the loading direction, another group penetrated the ring and the fracture surfaces across the center of the ring were generated in the vertical loading direction. It is found that there were no noticeable frictions and dislocations in the surfaces by observing the fracture surfaces of rock blocks after the tests. The ring structure can be recovered well in splicing of rock blocks and the fracture surfaces in the splicing position were better coupled. It can be concluded that two groups of tensile stress effect concentration zones were generated inside the ring granite under the radical compression or impact loads. One group expanded along the loading direction, another group expanded in the vertical loading direction, which was the main factor leading to the tensile failure in the ring granite.

By comparing and analyzing the failure modes of ring granite under the static compression loads and dynamic impact loads, it is easy to find that the ring specimens were broken in to two or four blocks. The failure modes of ring specimens with the smaller inner diameters were similar to the failure modes of Brazilian discs under uniaxial compression loads, i.e., the ring specimens were broken into two half rings across the center of the ring along the loading direction. However, the ring specimens with larger inner diameters were broken into four blocks with the similar fragment. Under the radical impact loads, the broken extent of ring specimens were more serious, part of ring specimens were broken into five blocks or more blocks. In addition, the crack position was not complete after splicing, and the two main fracture surfaces also deviated from the orthogonal direction. It could be explained that the static loading rate increased slowly and the increase rate kept flat, the damage cracks inside the ring granite orderly expanded along the tensile stress concentration direction. Under the impact loads, as the loading and unloading were completed instantly, the broken ring specimens were affected by the shock of the incident bar and transmitted bar, the dislocation and friction inevitably appeared, which led to the occurrence of the secondary cracks in the broken granite and the increasing number of rock blocks.

## Discussions on the failure criteria of ring granite structure

Based on the static and dynamic failure characteristics of ring granite under the water-heat cycle conditions, it is easy to find that the tensile failures are generated in the inner hole along the loading direction and in the outer ring wall in vertical loading direction. Finally, the ring specimens were broken into four blocks of approximately symmetric specimen fragments. Therefore, deducing the tensile stress equation in the micro cracks initiation position of ring specimens and combining with the static and dynamic tensile strength of ring granite could further predict the critical condition of failure in the ring specimens.

### Basic assumptions

The inner circle of ring structure directly affects the stress effect of each position inside the rock sample and the rock is geological material with non-uniformity, discontinuity, anisotropy and other properties, so discussions on the failure criteria of ring specimens under the static and dynamic radical loads should be based on the assumptions as follows.

1. It is assumed that the circular granite is homogeneous and the rocks are continuous and isotropic.
2. Assuming that under the radical compression, the tensile stress effect and tensile strain generated inside the ring specimens obey Hooke's Law.
3. Assuming that the tensile failure cracks generated in the ring granite expanded along the tensile stress concentration zone.
4. It is assumed that two groups of fracture surfaces generated in the ring granite pass through the ring center and are perpendicular to each other.
5. Assuming that the radial compressive stress is uniformly distributed in the contact line between the bearing plate and the ring specimen.

### Discussions on the criteria

**Figure 7:**
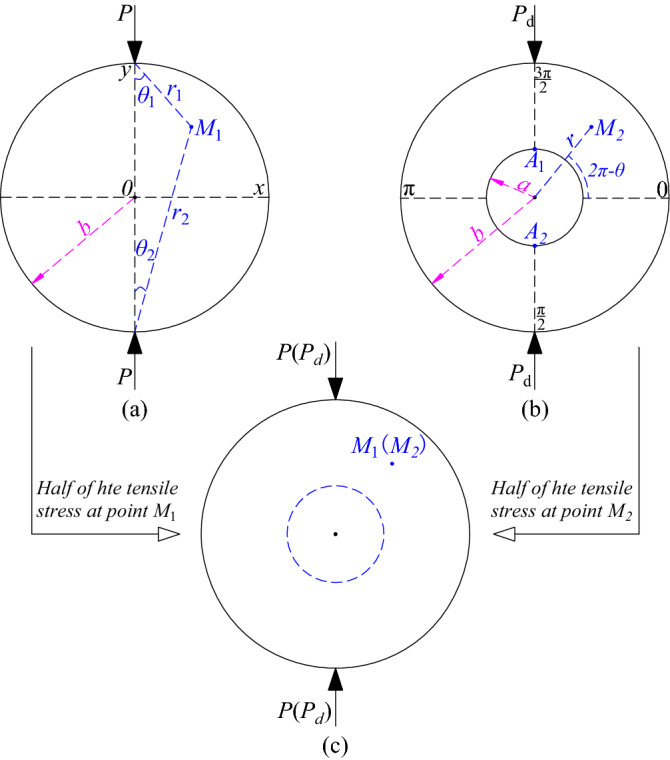
Schematic diagram of tensile stress deduction mechanics at any point in rock sample.

Based on the assumptions (1–5), the elasticity theory was used to deduct the tensile stress of ring granite passing the ring center line along the loading direction, which refers to the tensile stress at point *A*_*1*_ and point *A*_*2*_. Then, the corresponding criteria were attained via comparing the tensile stress deduced based on the equation with the static and dynamic tensile strength of granite. The specific diagram of mechanic calculations is shown in Fig. 7.

From Fig. 7a, the stress at any point in the Brazilian disc under the radical compression loads is obtained via the Boussinesq solutions and expressed as follows^31,32^:

2 $$ \left\{ {\begin{array}{*{20}l} {\sigma_{x} = \frac{2P}{{{\uppi }h}}\left( {\frac{{\sin^{2} \theta_{1} \cos \theta_{1} }}{{r_{1} }} + \frac{{\sin^{2} \theta_{2} \cos \theta_{2} }}{{r_{2} }}} \right) - \frac{P}{{{\uppi }Rh}} } \\ {\sigma_{y} = \frac{2P}{{{\uppi }h}}\left( {\frac{{\cos^{{3}} \theta_{1} }}{{r_{1} }} + \frac{{\cos^{{3}} \theta_{2} }}{{r_{2} }}} \right) - \frac{P}{{{\uppi }Rh}}} \\ {\tau_{xy} = \frac{2P}{{{\uppi }h}}\left( {\frac{{\cos^{2} \theta_{1} \sin \theta_{1} }}{{r_{1} }} - \frac{{\cos^{2} \theta_{2} \sin \theta_{2} }}{{r_{2} }}} \right)} \\ \end{array} } \right. $$

where* P* is the radical load that ring specimen could bear, *h* is the height of the ring specimen, *R* is the outer diameter of the ring specimen, i.e., *b* in Fig. 7a.

Letting *θ*_1_ = *θ*_2_ = 0, *r*_1_ = (*b*-*a*) , *r*_2_ = (*b* + *a*) , the formula (3) of the stress calculation at the point *A*_*1*_, *A*_*2*_ in Fig. 7a can be attained from formula (2).

3 $$ \left\{ {\begin{array}{*{20}l} {\sigma_{x} = - \frac{P}{{{\uppi }Rh}}\begin{array}{*{20}l} {\begin{array}{*{20}l} {} & {} & {} \\ \end{array} } & {} \\ \end{array}} \\ {\sigma_{y} = \frac{2P}{{{\uppi }h}}\left( {\frac{1}{b - a} + \frac{1}{b + a}} \right) - \frac{P}{{{\uppi }Rh}}\mathop {}\limits^{{}} } \\ {\tau_{xy} = 0 \begin{array}{*{20}l} {} & {} & {} \\ \end{array} } \\ \end{array} } \right. $$

According to Fig. 7b, by removing the parts where the power series are more than 2, the tensile stress at the point *A*_*1*_, *A*_*2*_ in the ring wall is approximate expressed as follows^33^:

4 $$ \begin{aligned} \sigma_{{{\text{r}} = {\text{a}}}} & = \frac{P}{\pi bh}\left[ { - \frac{2}{{1 - q^{2} }}} \right] \\ & \quad + \frac{2P}{{\pi bh}}\sum\limits_{n = 2}^{\infty } {\frac{{\left( { - 1} \right)^{n/2} 2nq^{n - 2} \left( {1 - q^{2} } \right)\left( {1 - q^{2n} } \right)\cos n\theta }}{{\left( {1 - q^{2n} } \right) - n^{2} q^{2n - 2} \left( {1 - q^{2} } \right)^{2} }}} \\ \end{aligned} $$

Letting *q* = *a*/*b*, *θ* = *π*/2 or 3*π*/2 in formula (4), the tensile stress at the point *A*_*1*_, *A*_*2*_ in the ring wall can be obtained from formula (5), as follows:

5 $$ \sigma_{{{\text{r}} = {\text{a}}}} = \frac{P}{\pi bh}\left[ {6 + 38\left( \frac{a}{b} \right)^{2} } \right] $$

Formula (3) is derived from the semi-infinite body subjected to the concentrated stress and formula (5) is applied to calculating the strength in the ring specimens with smaller inner diameters. If the formulas were employed in discussions on the failure criteria of ring granite, it might result in the bigger error. Based on static and dynamic test data of ring granite under the water-heat cycle conditions, it was found by the trial that the average value between the value calculated by formula (3) and formula (5) to predict the failure condition is a reasonable choice. In addition, the tensile strength of ring granite can be measured in the test based on the assumption (2). In conclusion, comparing the theoretical value of the tensile stress in the inner ring wall of ring granite along the loading direction with the value attained in the tests is able to predict the moment when ring specimens are broken. The criteria formulas are expressed as follows:

6 $$  \left\{ {\begin{array}{*{20}l} {\frac{P}{\pi bh}\left[ {\frac{7}{2} + 19\left( \frac{a}{b} \right)^{2} } \right]E\varepsilon } \hfill & {\left( {Broken} \right)} \hfill \\ {\frac{P}{\pi bh}\left[ {\frac{7}{2} + 19\left( \frac{a}{b} \right)^{2} } \right] = E\varepsilon } \hfill & {\left( {Critical \, state} \right)} \hfill \\ {\frac{P}{\pi bh}\left[ {\frac{7}{2} + 19\left( \frac{a}{b} \right)^{2} } \right]E\varepsilon } \hfill & {\left( {Steady} \right)} \hfill \\ \end{array} } \right. $$

### Test verification

**Figure 8:**
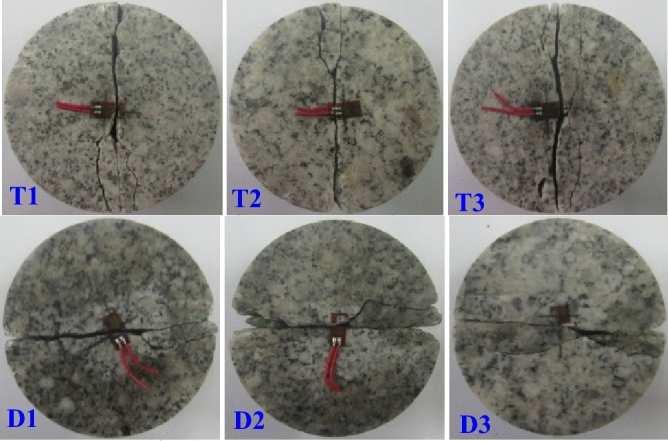
Static and dynamic cleavage failure modes of Brazilian disc.

In order to explore the rationality of the failure criterion of circular granite structure proposed above, two groups of experimental data under the radical static compression loads and impact loads were respectively chosen to verify the relationship between the theoretical value of tensile stress in the inner hole along the loading direction and the tensile strength of ring granite when the ring specimens are broken. The static and dynamic tensile elastic modulus and the tensile strain in the crack initiation were detected by sticking the strain gages in the center of a Brazilian discs, the broken ring specimens after the tests and parameters detected in the tests are shown in Fig. 8 and Table 2, respectively.

**Table 2:** Experimental results of tensile strength parameters of granite.

Sample number	High temperature Treatment/°C	Curing Temperature/°C	Static Stretch elasticity modulus/GPa	Static Crack initiation strain/10^–3^	Dynamic Stretch elasticity modulus/GPa	Dynamic Crack initiation strain/10^–3^
T1	400	40	8.12	0.40		
T2			6.78	0.49		
T3			7.94	0.38		
D1	400	40			18.13	1.39
D2					20.69	1.27
D3					17.28	1.44

**Figure 9:**
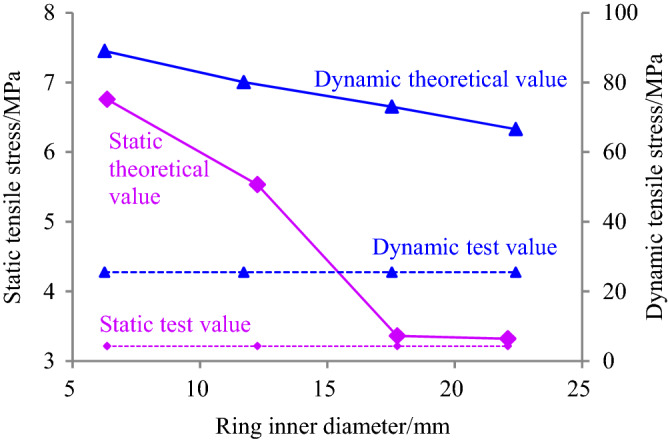
Verification diagram of static and dynamic failure criteria of circular granite.

By substituting the static and dynamic peak loads of ring granite subjected to high-temperature treatment at 400 °C and water curing at 40 °C and tensile parameters of ring granite measured through the tests in Table 2 into formula (6) and the corresponding static and dynamic theoretical and experimental values are deduced. By contrast, the analysis results are shown in Fig. 9.

In Fig. 9, the position to be verified was the inner ring wall of ring granite. It is seen clearly that under the static and dynamic radical loads, the tensile stress attained by the failure criteria in the inner ring wall along the loading direction was larger than the tensile strength of ring granite measured by sticking the strain gages in the center of a Brazilian discs. It means that under the static and impact radical loads, the tensile stress effect that the inner ring wall undertook reached the critical station of crack initiation along the loading direction at a certain moment before the peak load, i.e., cracks initiated from the inner ring wall at a certain moment before the peak load. Therefore, the initial cracks were generated in the inner ring wall along the loading direction. Then the cracks initiated from the inner ring wall and penetrated the ring specimen. During this period, the macro-failure occurred. Furthermore, in the water-heat cycle experiment, damage cracks first initiated from the inner ring wall along the loading direction under the static and dynamic radical compression loads. By contrast, it is found that the verification results and experimental results are consistent. Therefore, it proves that static and dynamic failure criteria of ring structure are reasonable.

## Conclusions

Based on the engineering environment of wellbore surrounding rock in the energy storage area during hydrothermal geothermal energy exploitation, experimental study on static and dynamic failure characteristics of circular granite under hydrothermal cycling condition were carried out. The specific conclusions are drawn as follows:

1. Under the various factors, the load-strain curves of ring granite under the impact loads were nonlinear. However, the changes of the curves under static radical loads were more influenced by the environment and shown as the concave stages, straight stages, plat stages, nonlinear stages, etc.
2. Under the static and dynamic radical loads, the peak loads of ring specimens decreased exponentially with the growth of damage factors and the dynamic peak loads were far greater than the static peak loads.
3. The apparent deformation modulus was defined to describe the deformation characteristics of ring granite. It is found that the static apparent deformation modulus decreased and the changes of dynamic apparent deformation modulus were more dispersed with the growth of damage factors.
4. It is found that static and dynamic damage cracks were tensile cracks. The dynamic damage cracks expanded rapider and the crack spacing was larger. In addition, the broken degree of ring specimens under radical impact loads was more serious than that under static loads, but the ring structure could be recovered well in splicing of rock blocks and the fracture surfaces in the splicing position were better coupled, indicating that the final failure mode of ring granite was tensile failure.
5. Based on the deformation and failure characteristics of circular granite and the inner wall of the ring granite was taken as the analysis position, the static and dynamic failure criteria were deduced and verified by the corresponding tests.

## Data Availability

The datasets generated during and/or analysed during the current study are available from the corresponding authors on reasonable request.
